# Correlation between target volume and electron transport effects affecting heterogeneity corrections in stereotactic body radiotherapy for lung cancer

**DOI:** 10.1093/jrr/rrt231

**Published:** 2014-02-11

**Authors:** Yuichi Akino, Indra J. Das, Higinia R. Cardenes, Colleen M. Desrosiers

**Affiliations:** Department of Radiation Oncology, Indiana University School of Medicine, 535 Barnhill Drive RT 041, Indianapolis, IN 46202, USA

**Keywords:** heterogeneity corrections, lung cancer, stereotactic body radiotherapy, radiation dosimetry

## Abstract

Recently, stereotactic body radiotherapy (SBRT) for lung cancer is conducted with heterogeneity-corrected treatment plans, as the correction greatly affects the dose delivery to the lung tumor. In this study, the correlation between the planning target volume (PTV) and the dose delivery is investigated by separation of the heterogeneity correction effects into photon attenuation and electron transport. Under Institutional Review Board exemption status, 74 patients with lung cancer who were treated with SBRT were retrospectively evaluated. All treatment plans were generated using an anisotropic analytical algorithm (AAA) of an Eclipse (Varian Medical Systems, Palo Alto, CA) treatment planning system. Two additional plans were created using the same treatment parameters (monitor units, beam angles and energy): a plan with no heterogeneity correction (NC), and a plan calculated with a pencil beam convolution algorithm (PBC). Compared with NC, AAA and PBC isocenter doses were on average 13.4% and 21.8% higher, respectively. The differences in the isocenter dose and the dose coverage for 95% of the PTV (*D*_95%_) between PBC and AAA were correlated logarithmically (*ρ* = 0.78 and *ρ* = 0.46, respectively) with PTV. Although *D*_95%_ calculated with AAA was in general 2.9% larger than that for NC, patients with a small PTV showed a negative Δ*D*_95%_ for AAA due to the significant effect of electron transport. The PTV volume shows logarithmic correlation with the effects of the lateral electron transport. These findings indicate that the dosimetric metrics and prescription, especially in clinical trials, should be clearly evaluated in the context of target volume characteristics and with proper heterogeneity correction.

## INTRODUCTION

Stereotactic body radiotherapy (SBRT) has gained worldwide acceptance as treatment for early stage inoperable small lesions of non-small cell lung cancers (NSCLCs), and has yielded excellent tumor control rates, above 90% [1, 2]. Several clinical trials have been conducted to investigate the use of SBRT for NSCLC treatment. Radiation Therapy Oncology Group (RTOG) protocols 0236 and 0618 did not allow heterogeneity corrections for dosimetry and monitor unit calculation because of a lack of clinical experience in heterogeneity correction. Later, RTOG protocols 0813 and 0915 did require heterogeneity corrections in dose calculation [3]. Dose calculation in lung SBRT is complicated as a consequence of the dosimetry of thoracic organs with tissue heterogeneities and small treatment fields [4]. The accuracy of dosimetry has improved a great deal, with advanced commercial dose calculation algorithms such as collapsed-cone, convolution/superposition, anisotropic analytical algorithm (AAA), Acuros^®^ XB and Monte Carlo [5–9]. The Japan Clinical Oncology Group (JCOG) 0403 phase II trial and 0702 phase I trial were conducted using heterogeneity correction with one-dimensional equivalent path length (EPL) and a convolution/superposition algorithm, respectively [10]. Recently, some clinical trials have been conducted using advanced dose calculation algorithms.

Radiation dosimetry plays an important role in the comparison of clinical outcome. In light of this, the impact of heterogeneity corrections on dose calculation in lung SBRT has been investigated [11–14]. Many of the earlier studies that investigated the effect of heterogeneity corrections using clinical data assessed a small number of cases or collected patient data from multiple institutions. Xiao *et al*. [15] and Schuring and Hurkmans [16] reported that the dose covering 95% of the PTV (*D*_95%_) for a PTV calculated with superposition or collapsed-cone was smaller than that for a PTV calculated without heterogeneity corrections. Recently, Ueki *et al*. [17] investigated 83 SBRT plans and reported the opposite for *D*_95%_ results. In their study, the *D*_95%_ of a PTV calculated with AAA was slightly larger than that calculated without corrections, however they did not mention the discrepancy between previous reports and their study. The effect of heterogeneity corrections on target coverage is critical when determining treatment protocols. Although previous studies evaluated the average ± SD of the dose–volume metrics, or statistical differences among various dose calculation algorithms, few studies have investigated the factors that affect the heterogeneity corrections.

To evaluate more detailed effects of heterogeneity corrections with advanced algorithms on the target dose of SBRT for lung cancer, a retrospective analysis of the treatment plans of 74 patients was conducted at a single institution. The results were analyzed according to size of target volume and by separating photon attenuation and electron transport for the heterogeneity corrections.

## MATERIALS AND METHODS

### Patients

Under Institutional Review Board (IRB) exemption, we retrospectively analyzed the treatment plans of 74 patients with lung cancer who were treated with SBRT at Indiana University School of Medicine, Indianapolis. There were 22 and 52 cases in the right- and left-lobe of the lung, respectively. Details of the prescribed doses for these patients are listed in Table 1. Every SBRT patient was immobilized using either an Elekta Stereotactic Body Frame (Elekta, Stockholm, Sweden) or a CIVCO Body Pro-Lok system (CIVCO, Kalona, IA), depending upon the patient size, comfort and suitability as decided at the time of simulation.

**Table 1.** Description of treatment parameters

Plan parameters	Frequency
Energy	
6 MV	50 (68%)
6 + 16 MV^a^	24 (32%)
Number of beams	
8	3 (4%)
9	15 (20%)
10	49 (66%)
11	7 (10%)
Number of non-coplanar beams	
≤2	2 (3%)
4	18 (24%)
5	46 (62%)
6	8 (11%)

^a^6 + 16 MV = treatment fields including both 6 MV and 16 MV beams.

### Treatment planning and evaluations

Eclipse version 10.0 was used for the treatment planning. Plans were generated to cover 95% of the PTV with the prescribed dose, thus providing typically a 25% higher dose to the GTV. Eclipse provides two algorithms for heterogeneity correction; pencil beam convolution (PBC) and AAA. The PBC model in Eclipse is mainly for homogenous medium (water) dose calculation that gets supplemented with older models modified Batho, Batho power law and equivalent tissue air ration (ETAR) [18]. It was noted that both modified Batho and Batho power law gave almost the same results for SBRT, and hence default modified Batho with PBC was used. The ETAR option with PBC cannot be used with non-coplanar field arrangements, thus ETAR was not an option in this study as the majority of the SBRT fields were non-coplanar. Heterogeneity correction was applied to all clinically approved plans using an AAA algorithm. The AAA is considered a superior algorithm compared with older and pencil beam algorithms [7, 19–21]. Two additional plans were generated for each patient: (i) a treatment plan with no correction (NC), and (ii) PBC with modified Batho heterogeneity correction. In the PBC algorithm, the dose deposited at a point was calculated as a convolution of energy fluence, or total energy released per unit mass (TERMA), with the respective dose deposition kernel pre-calculated for a narrow beam in water [22]. The Batho power-law correction method is an empirical correction to account for both primary beam attenuation and scatter changes in heterogeneous materials. The modified Batho correction uses only the descending part of the TAR/TMR curve because the curve in the build-up region of a high-energy photon is no longer valid. However, PBC does not take into account changes in lateral electron transport. This provided us with an opportunity to differentiate between photon attenuation (PBC) versus electron transport (AAA).

For each patient, two additional plans (NC and PBC) were created using the same monitor units as calculated with AAA. Because PBC considers changes in tissue density but does not consider lateral electron transport, the difference between the NC and PBC can be considered as the effect of photon attenuation. As AAA considers both depth correction and electron transport, the difference between PBC and AAA therefore represents the effect of electron transport calculation.

To evaluate the effect of photon attenuation, we assessed the change in path length (ΔPL) defined as:

(1) $$\Delta PL=\sum_{i}\left(d_{i}-EPL_{i}\right)\frac{W_{i}}{W}$$

where *d*_*i*_ and *EPL*_*i*_ represent the physical and equivalent path length (EPL) depth for each treatment beam, respectively. *W*_*i*_ and *W* represent the weight of each beam and the total weight, respectively. The *D*_95%_ and the volume receiving the prescribed dose (*V*_100%_), representing the quality of the target volume coverage, were also analyzed.

### Statistical analysis

JMP software (ver. 9.0.2; SAS Institute, Cary, NC) was used for statistical analysis. All pairwise comparisons among the three calculation algorithms were conducted for the isocenter dose (*D*_Iso_), *D*_95%_ and *V*_100%_ using the Steel–Dwass test. The correlation between dosimetric parameters and anatomical characteristics including ΔPL, the PTV and the distance between the PTV and the chest wall or mediastinum was assessed with Spearman's rank correlation coefficient. Statistical significance was defined as a *P-*value < 0.05.

## RESULTS

**Fig. 1.**
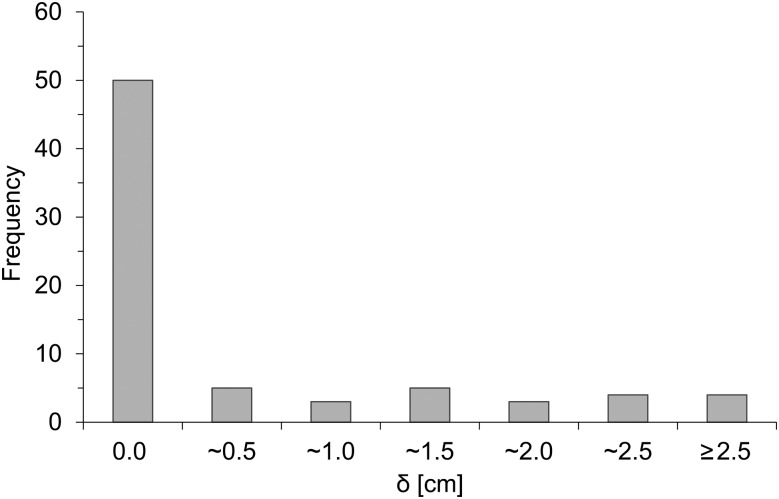
Frequency distribution of the shortest distance (δ) between gross tumor volume (GTV) and chest wall or mediastinum.

**Fig. 2.**
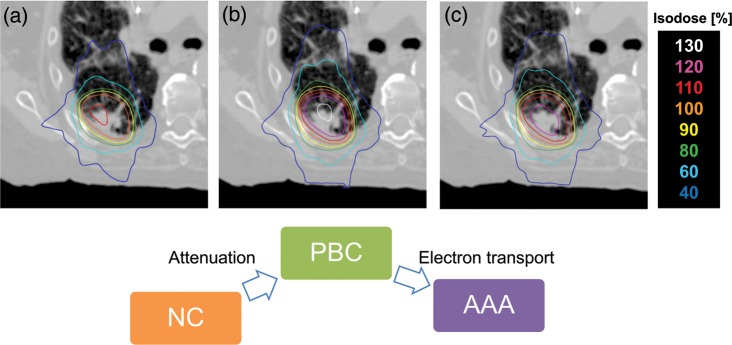
Axial dose distributions of a patient calculated using (**a**) no heterogeneity correction (NC), heterogeneity correction using (**b**) PBC with modified Batho, and (**c**) AAA.

The characteristics of target volumes of the patients are shown in Table 2. Due to the selection criterion in our institution, peripheral lesions were preferred for SBRT, as shown in Fig. 1, which indicates the frequency distribution of lesions. This figure shows that the majority of the patients (74.3%) have the GTV close to the chest wall (<0.5 cm) and the remainder have it distributed evenly up to 2.5 cm. Figure 2 shows an example of dose distributions using the three treatment plans (NC, PBC, AAA). The subtle dose difference is clearly visible. Typically, PBC generally overestimates dose in lung as it underestimates the range of the secondary particles, whereas AAA provides accurate dose distribution.

**Table 2.** Details of target volume

Anatomic parameters	Mean ± SD (min − max)
Target volume	
GTV	13.8 ± 16.5 (0.6 − 111.5) [cm^3^]
PTV	45.3 ± 35.7 (9.5 − 212.0) [cm^3^]
Maximum dimension	
GTV	3.34 ± 1.31 (1.26 − 7.02) [cm]
PTV	5.12 ± 1.33 (1.54 − 8.75) [cm]

GTV = gross tumor volume, PTV = planning target volume.

### Isocenter dose

**Fig. 3.**
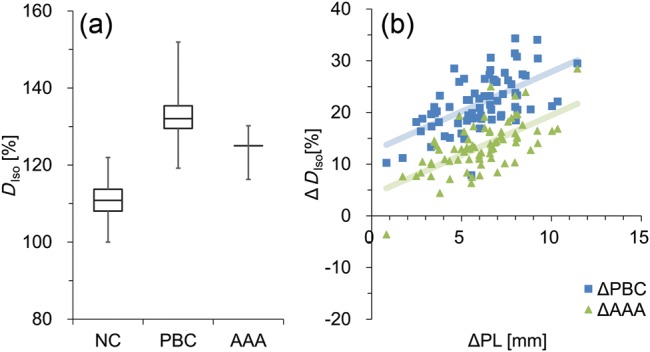
(**a**) The isocenter dose (*D*_Iso_) calculated without (NC) and with (PBC with modified Batho and AAA) heterogeneity corrections are shown. Boxes represent median and quartile values. Whiskers represent maximum and minimum values. (**b**) The differences between *D*_Iso_ calculated without and with heterogeneity corrections (ΔD_Iso_) are illustrated.

The *D*_Iso_ relative to the prescribed dose for each plan is shown in Fig. 3a. The *D*_Iso_ (average ± SD) for the entire patient population was 110.8 ± 4.4%, 132.6 ± 4.3% and 124.2 ± 3.1% for NC, PBC and AAA, respectively. The Δ*D*_Iso_ calculated with PBC and AAA were larger than that calculated with NC by 21.8% and 13.4%, respectively, in our population, with both *P* < 0.0001. The *D*_Iso_ of AAA was 8.4% smaller than that of PBC (*P* < 0.0001). In Fig. 3b, the isocenter dose difference (Δ*D*_Iso_) between the dose with and without heterogeneity corrections of PBC (ΔPBC) and AAA (ΔAAA) were plotted with ΔPL as shown in Eq 1. The slope of the lines for PBC and AAA are nearly identical. The ΔPBC and ΔAAA showed intermediate linear correlation with ΔPL (*ρ* = 0.60, *P* < 0.0001 for both).

### Target coverage

**Fig. 4.**
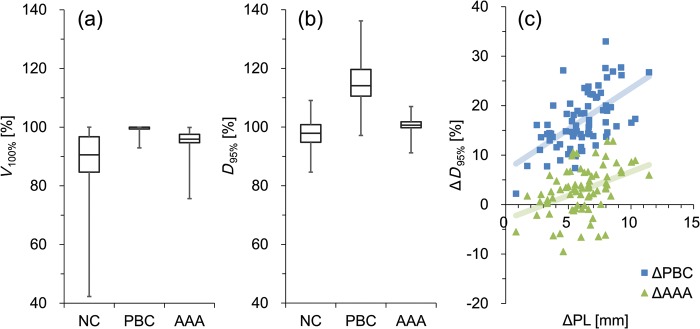
(**a**) The volume receiving the prescribed dose (*V*_100%_), and (**b**) the dose covering the 95% PTV (*D*_95%_) calculated without (NC) and with (PBC with modified Batho and AAA) heterogeneity corrections. Boxes represent median and quartile values. Whiskers represent maximum and minimum values. (**c**) The difference between the *D*_95%_ calculated without and with heterogeneity corrections (Δ*D*_95%_) are illustrated.

Typically, patients are treated with better than 95% target coverage, as shown in Fig. 4a indicating *V*_100%_ with NC, PBC and AAA algorithms for all patients. The median *V*_100%_ was 90.6% (range, 42.3–98.7%), 100.0% (range, 92.9–100.0%) and 96.0% (range, 75.6–99.9%) for NC, PBC and AAA, respectively. The long error bars indicate the variability among the patients. The average ± SD of *D*_95%_ was 97.6 ± 5.0%, 114.6 ± 7.4% and 100.5 ± 2.8% for NC, PBC and AAA, respectively as shown in Fig. 4b. The *D*_95%_ of PBC and AAA were larger than that of NC by 17.0% (*P* < 0.0001) and 2.9% (*P* < 0.0001), respectively. The Δ*D*_95%_ of AAA plans was 14.1% smaller than that of PBC (*P* < 0.0001). Figure 4c illustrates the evaluation of Δ*D*_95%_ of the PTV for each patient, representing a similar analysis to that of Fig.3b for *D*_Iso_. The ΔPBC shows intermediate linear correlation with ΔPL (*ρ* = 0.58, *P* < 0.0001), although ΔAAA showed weaker correlation with ΔPL (*ρ* = 0.41, *P* = 0.0003).

### Correlation with target volume

**Fig. 5.**
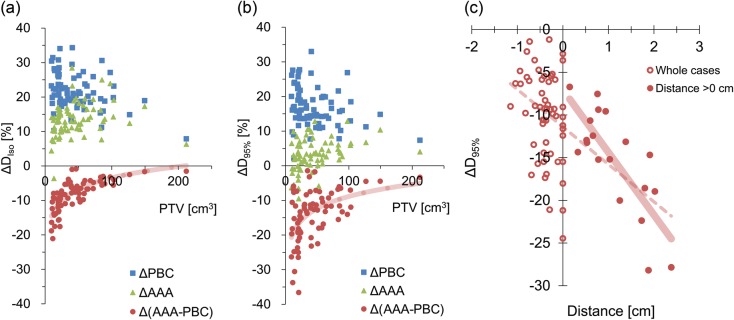
(**a**) Δ*D*_Iso_ and (**b**) Δ*D*_95%_ between the doses without and with heterogeneity corrections were plotted for ΔPBC and ΔAAA. The dose differences between PBC and AAA are plotted with the PTV volume of each case. Lines represent the logarithmic approximation for Δ(AAA − PBC). (**c**) Differences in *D*_95%_ between PBC and AAA are plotted against the shortest distance between the PTV and the chest wall or mediastinum. Negative distance represents the largest overlapped distance between the PTV and the chest wall. Dashed and solid lines represent linear approximations of whole cases and cases with the distance >0 cm, respectively.

The size of the PTV is correlated with Δ*D*_Iso_ and Δ*D*_95%_ between the dose with and without heterogeneity corrections, as shown in Fig. 5a and b, respectively. The dose differences between PBC and AAA Δ(AAA − PBC), are also plotted. Interestingly, the Δ*D*_Iso_ of Δ(AAA − PBC) showed significant logarithmic relationship with the PTV volume (*ρ* = 0.78, *P* < 0.0001). Almost all ΔAAA cases showed positive Δ*D*_Iso_, although one patient showed a negative value. This result indicates that the effect of attenuation was larger than that of electron transport for most cases. As illustrated in Fig. 5b, the Δ*D*_95%_ of Δ(AAA − PBC) showed intermediate logarithmic correlation with the PTV volume (*ρ* = 0.46, *P* < 0.0001), although the result showed a larger deviation than that of Δ*D*_Iso_. Most cases of ΔAAA showed a positive value of Δ*D*_95%_, but for a small PTV volume it showed negative values, probably as a consequence of significant effects of electron transport. In Fig. 5c, the Δ*D*_95%_ of Δ(AAA − PBC) were plotted against the shortest distance between the PTV and the chest wall or mediastinum. Negative distance represents the largest overlapped distance between the PTV and the chest wall. Although isolated tumors with positive distance showed moderate linear correlation with the distance (*r*^2^ = 0.62, *P* < 0.0001), whole cases, including tumors attached to chest wall, showed weaker correlation (*r*^2^ = 0.34, *P*<0.0001).

**Fig. 6.**
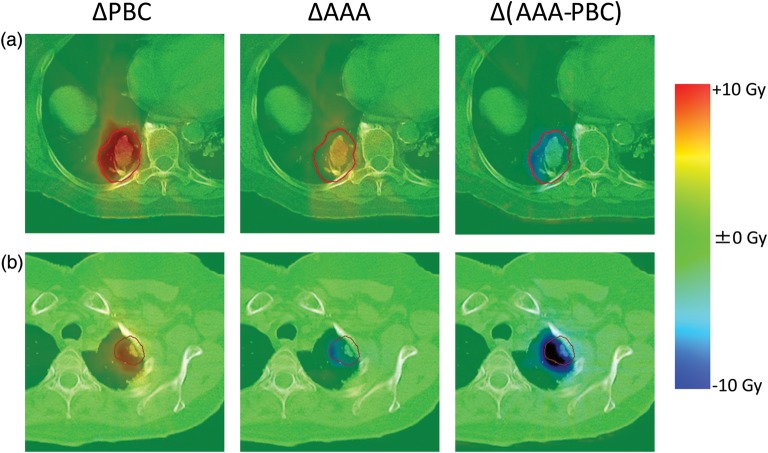
Differences in dose distribution for ΔPBC, ΔAAA and Δ(AAA − PBC). The representative patients with (**a**) large, and (**b**) small tumors are illustrated.

In Fig. 6, the differences in the dose distribution have been illustrated for representative patients with (a) large (102.4 cm^3^) and (b) small (13.2 cm^3^) PTV volumes. The ΔPL were 3.5 cm and 0.8 cm for patients (a) and (b), respectively. Patient (a) showed a larger attenuation effect in the ΔPBC as a consequence of a large lung:solid tissue ratio in the path length. In the Δ(AAA − PBC) of patient (b), the larger lateral electron transport effect is visualized, resulting in the negative net effect shown in ΔAAA.

## DISCUSSION

Several studies have investigated the impact of heterogeneity corrections on the dose distribution in lung SBRT with similar findings. We demonstrated that the *D*_95%_ of a PTV calculated with AAA is 2.9% larger than that of a PTV calculated with NC (Fig. 4b). Interestingly, this is the opposite of *D*_95%_ results reported by Xiao *et al*. (−7.6%) and Schuring and Hurkmans (− 5.2%), although our results of *D*_Iso_ (13.4%) were similar to the previous report by Xiao *et al*. (12.5%) [15, 16]. Recently Ueki *et al*. investigated 83 patients and reported that the PTV *D*_95%_ under AAA calculation was higher than that without corrections by ∼1.4% [17]. Their results for *D*_95%_ were similar to ours, although they did not investigate the factors that affect the heterogeneity corrections.

We demonstrated the significant logarithmic relationship between the effect of lateral electron transport and PTV volume (Fig. 5a and b). Cases with a large PTV showed smaller effects from the lateral electron transport than those from attenuation, resulting in the positive Δ*D*_95%_ of ΔAAA. In contrast, with a small PTV, the effects of electron transport become larger (Fig. 6b). Although the average PTV *D*_95%_ of AAA plans is 2.9% larger than that of NC, it depends on the anatomical characteristics of the patients analyzed in this study. Schuring and Hurkmans previously investigated the effects of heterogeneity corrections on the dose distribution of 26 SBRT patients and reported that the collapsed-cone convolution algorithm showed decreased conformity with the PTV volume [16]. On the other hand, van der Voort van Zyp *et al*. compared EPL and the Monte Carlo algorithm with respect to tumor location and size [23]. They separated patients roughly by tumor size and reported that the difference in *D*_95%_ between two algorithms was larger for small tumors. The results of previous studies that investigated various calculation algorithms cannot be simply compared without considering the variation in target volume characteristics. The lateral electron transport is affected by the medium density, size of the target volume and also by the surrounding tissue, such as chest wall. Narabayashi *et al*. [24] investigated the lung SBRT plans for 20 patients and reported that the ratio of MU values calculated with the Batho Power Law and Monte Carlo showed linear correlation with the distance between the PTV and the chest wall. We also analyzed the correlation between the effects of lateral electron transport and the distance between the PTV and the chest wall or mediastinum (Fig. 5c). Although the data of isolated tumors showed moderate linear correlation, the whole cases (including tumors attached to the chest wall) showed weaker correlation. To properly assess the effect of the surrounding tissue on the lateral electron transport, the area of attachment to the surrounding tissue, and the volume of the chest wall included in the PTV (and its density) should also be considered. These complexities of the lateral electron transport effects caused by the surrounding tissue will lead to larger *D*_95%_ variation of Δ (AAA − PBC), illustrated in Fig. 5b.

In the current study, the treatment plans were clinically optimized based on AAA; and the beam arrangements, energy and MU were not modified when calculating with NC and PBC. The differences in dosimetric parameters between AAA and the other two algorithms become smaller with optimizing plans, although the results include the uncertainty due to the individual planning techniques. Some studies compared commercial Monte Carlo dose calculation with other algorithms for dose calculation of lung SBRT [3, 25, 26]. Li *et al*. noted that some of the criteria of treatment protocols for superposition might be too strict for the Monte Carlo calculation to satisfy the requirements [3]. Several previously reported that the advanced dose calculation algorithm showed worse target coverage. However, we showed in this study that the target coverage is strongly affected not only by calculation algorithms but also by the anatomic characteristics of the patients.

We demonstrated the effects of the heterogeneity correction by separating them into the attenuation and lateral electron transport components. The PBC provides photon attenuation, whereas AAA accounts for the electron transport that is needed for lung SBRT. The PTV volume shows significant correlation with the effects of the lateral electron transport with logarithmic correlation. When the patients are not classified with target volumes, the dosimetric parameters evaluated with average ± SD may not be able to assess the effects of the heterogeneity corrections properly. Several clinical trials for lung SBRT are being conducted using the target coverage-based prescription with heterogeneity corrections. It is concluded, therefore, that when radiation outcome is compared between clinical trials, dosimetric metrics, the target volume characteristics and heterogeneity correction algorithms should be properly accounted.

## FUNDING

This work was supported by the Japan Society for the Promotion of Science (JSPS) Core-to-Core Program (No. 23003) and a research grant from Varian Medical Systems (No. 4513228).
